# Determinants of Patient Satisfaction in a Cardiac Telemedicine Clinic: A Cross-Sectional Study From Saudi Arabia

**DOI:** 10.7759/cureus.101613

**Published:** 2026-01-15

**Authors:** Ahmed M Badheeb, Hadi S Alyami, Nada M Al Jaman, Ali A Al Manajam, Abdulhadi M Alyami, Sarah Al Qahtani, Ibrahim Hassan, Yahya Alhosni, Abdulhakim Noman, Ehab Ali, Mohammed Al-Shikh, Abubaker A Mohammed, Thabit Mohamed, Yazan Hassan, Mohamed Badheeb

**Affiliations:** 1 Oncology Center, King Khalid University Hospital, Najran, SAU; 2 Epidemiology and Public Health Department, General Directorate of Health Affairs, King Khalid University Hospital, Najran, SAU; 3 Nursing Department, King Khalid University Hospital, Najran, SAU; 4 Operating Room, King Khalid University Hospital, Najran, SAU; 5 Cardiology Department, Prince Sultan Cardiac Center, Najran, SAU; 6 Cardiology Department, King Khalid University Hospital, Najran, SAU; 7 Medicine Department, King Khalid University Hospital, Najran, SAU; 8 Internal Medicine Department, Yale New Haven Health, Bridgeport Hospital, Bridgeport, USA

**Keywords:** cardiac telemedicine, chronic disease, digital health, health equity, najran, patient satisfaction, saudi arabia, virtual clinic

## Abstract

Background

The rapid integration of telemedicine into cardiovascular care requires a comprehensive understanding of patient satisfaction to maintain service quality and equity. Although telemedicine adoption is increasing in Saudi Arabia, patient-centered evaluations, particularly for specialized services such as cardiology in non-urban regions, are lacking. This study assessed patient satisfaction and identified its key predictors within a cardiac telemedicine service in Najran, Saudi Arabia.

Patients and methods

A prospective cross-sectional study was conducted among 109 consecutive patients attending the Najran Cardiac Virtual Clinic between June and August 2024. Participants completed a validated electronic survey that collected sociodemographic data, clinical history, and satisfaction levels across 12 domains of telemedicine care, rated on a four-point Likert scale from Poor to Excellent. High overall satisfaction was defined as a composite score at or above the 75th percentile of the sample distribution. Predictors of satisfaction were identified using univariate and multivariable logistic regression models.

Results

The mean age of participants was 47.1±17.2 years, and 60 (55.0%) were female. Overall satisfaction was very high, with 104 patients (95.4%) rating their experience as "Good" or "Excellent." The highest-rated domains were confidentiality and privacy (104/109, 95.4% Good/Excellent) and ease of use (104/109, 95.4%). Multivariable analysis identified two independent predictors of high satisfaction: higher educational attainment (associated with 3.1 times higher odds of high satisfaction; adjusted odds ratio (aOR): 3.1, 95% CI: 1.2-8.0; p=0.019) and a lower count of comorbidities (fewer than three versus three or more comorbidities; aOR: 2.8, 95% CI: 1.1-7.3; p=0.034).

Conclusion

Cardiac telemedicine services in Najran achieved high patient satisfaction and demonstrated broad acceptability across age, gender, and residential settings. This study provides one of the first patient-centered evaluations of a dedicated cardiac telemedicine service in a non-urban region of Saudi Arabia. However, significantly lower satisfaction was reported by patients with lower educational attainment and those with multiple comorbidities. To ensure equitable delivery, these groups require prioritized interventions such as tailored digital literacy support, simplified interfaces, and integrated care pathways.

## Introduction

The global healthcare landscape has experienced significant transformation due to the rapid integration of telemedicine, a process accelerated by the COVID-19 pandemic [1,2]. In cardiology, telemedicine serves as a critical tool for improving access to specialist care, facilitating remote monitoring, and maintaining continuity of care for patients with chronic cardiovascular conditions [3]. The associated benefits include reduced travel requirements, shorter wait times, and more efficient resource allocation within healthcare systems [2,4].

In Saudi Arabia, this digital transformation is central to the Vision 2030 framework, which promotes digital health solutions to enhance the quality, efficiency, and accessibility of care across the Kingdom's extensive geography [4,5]. The sustainable implementation of telemedicine, however, depends not only on clinical efficacy but also on end-user acceptance, with patient satisfaction being a key indicator of healthcare quality and a major determinant of continued service use, adherence, and outcomes [2,4].

Although international studies frequently report high patient satisfaction with telecardiology, these findings are not universally generalizable. Satisfaction is a multifaceted construct shaped by demographic, socioeconomic, clinical, technological, and cultural factors [3,6-9]. For instance, older age, lower educational attainment, and limited digital literacy are recognized barriers that can exacerbate a digital divide [10,11]. Furthermore, patients with greater clinical complexity, such as multiple comorbidities, may perceive telemedicine as less comprehensive due to the inherent limitations of remote physical examination [6].

Emerging evidence from Saudi Arabia reflects a growing but nuanced acceptance of telehealth. AlRadini et al. reported that 86% of patients at a major university hospital were satisfied with virtual consultations, despite noting limitations such as missed appointments and the lack of physical exams [12]. Similarly, Asiri et al. found that over 60% of healthcare practitioners in a Makkah hospital viewed virtual clinics favorably while highlighting persistent infrastructural and training challenges [11].

Despite this strategic emphasis and growing use, a significant evidence gap persists. Robust, data-driven studies evaluating patient-centered outcomes in the Saudi context remain scarce, with existing literature focusing more on feasibility and clinician perspectives [10,11]. This gap is particularly pronounced for specialized care contexts such as cardiology and for services in non-urban regions, where patient needs, resources, and perceptions may differ. Identifying determinants of satisfaction is crucial for tailoring services, allocating resources efficiently, and ensuring the digital health transformation does not exacerbate existing health inequities.

This study, therefore, aimed to assess the level and determinants of patient satisfaction with a cardiac telemedicine service at King Khalid Hospital in Najran, Saudi Arabia. Its primary objective was to measure patient satisfaction across key domains of the telemedicine experience. The secondary objective was to identify sociodemographic and clinical predictors of high overall satisfaction. By providing one of the first patient-centered evaluations of a dedicated cardiac telemedicine service in a non-urban Saudi region, this study seeks to generate evidence to inform the optimized and equitable expansion of such services.

## Materials and methods

Study design and setting

A prospective cross-sectional study was conducted at the virtual cardiac clinic of King Khalid Hospital in Najran, Saudi Arabia, over a three-month period from June to August 2024. The clinic utilizes the Ministry of Health’s Sehhaty platform to deliver video-based consultations to adult patients residing in both urban and rural areas of the Najran region [13]. This approach enabled the inclusion of a representative sample with diverse sociodemographic characteristics and varying levels of healthcare access.

Study population and sample size determination

All consecutive adult patients (aged 18 years or older) who attended scheduled virtual cardiac consultations at King Khalid Hospital in Najran between June and August 2024 were assessed for eligibility. A total of 182 patients were screened during the study period. Exclusion criteria included significant cognitive or communication impairment precluding valid consent or survey completion, refusal to participate, and technical failures that prevented completion of the consultation.

**Figure 1:**
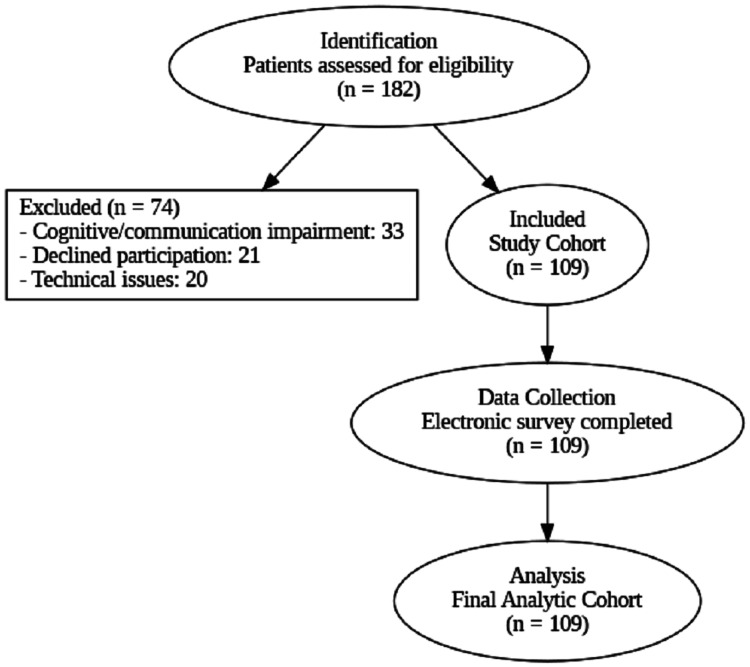
Participant flow diagram for the cardiac telemedicine satisfaction study.

An a priori power analysis, based on effect sizes from previous Saudi telemedicine studies [11], indicated that a sample size of 120 patients would provide 85% power to detect an odds ratio of 2.5 or greater for primary satisfaction predictors at a 0.05 significance level. Following application of the exclusion criteria, 74 patients were excluded: 33 due to cognitive or communication impairment, 21 due to declining participation, and 20 due to technical issues. The final analytic cohort consisted of 109 patients who completed the electronic satisfaction survey and were included in all statistical analyses (Figure 1). This sample size yielded approximately 80% statistical power for the multivariable regression models and represented the entire eligible cohort during the study period.

Data collection and instrument validation

Data were collected using a structured, self-administered electronic questionnaire, which was distributed by trained research assistants during or immediately after the virtual consultation. The instrument was adapted from validated telehealth satisfaction surveys used in prior Saudi studies [11]. The original instruments demonstrated high internal reliability (Cronbach's alpha: 0.82-0.91). Cultural adaptation included forward and backward translation and expert clinical review. Pilot testing with 15 patients produced a Cronbach's alpha of 0.87 for satisfaction domains, confirming strong internal consistency. Research assistants underwent standardized training to ensure unbiased and culturally sensitive administration, including the use of the Najrani Arabic dialect.

Variables and operational definitions

Sociodemographic variables comprised age, sex, nationality, marital status, education level (primary, secondary, university), employment status, and place of residence (urban or rural). Clinical variables included cardiac diagnosis (coronary artery disease, arrhythmia, or valvular disease, or none), comorbidities (hypertension, diabetes, obesity), and medication use. Polypharmacy, initially considered, was not included in the final analysis due to collinearity with comorbidity count and lack of significance in preliminary analyses.

Patient satisfaction was evaluated across 12 domains: privacy, voice clarity, visual clarity, physician courtesy, physician carefulness, treatment explanation, appointment speed, ease of connection, system reliability, personal comfort, response to login issues, and overall experience. Each domain was rated on a four-point Likert scale (Excellent = 4, Good = 3, Fair = 2, Poor = 1). A composite satisfaction score was calculated by summing the domain scores. High satisfaction was defined as a composite score at or above the 75th percentile of the sample's distribution, which corresponds to “Excellent” ratings in at least nine of the 12 domains, according to established telehealth benchmarks [14-16].

Technical quality was defined as follows: excellent voice clarity required the absence of audio distortion or echo, and excellent visual quality required no pixelation or freezing during the consultation.

Statistical analysis

Descriptive statistics were used to summarize participant characteristics and satisfaction ratings. Categorical variables were reported as frequencies and percentages, while continuous variables were presented as means with standard deviations or medians with interquartile ranges, as appropriate.

To identify predictors of high satisfaction, we first performed univariate logistic regression for all candidate variables. Variables with a univariate p-value < 0.20, along with those deemed clinically relevant (age, gender), were entered into an initial multivariable logistic regression model. A backward elimination process was then employed to retain variables significant at p < 0.05 in the final model. Multicollinearity was assessed using variance inflation factors (VIFs < 3). Missing data were minimal (less than 5% per variable) and were addressed using multiple imputation with five iterations to preserve statistical power and reduce bias. A sensitivity analysis comparing these results with a complete-case analysis confirmed the robustness of the primary findings.

Subgroup comparisons of mean satisfaction scores by age, education, and residence were performed using analysis of variance (ANOVA) with Tukey post-hoc tests. Multivariable linear regression was used to adjust for confounders, including sex, employment, comorbidity, and diagnosis. Statistical significance was defined as p < 0.05 (two-tailed). All analyses were conducted using Statistical Product and Service Solutions (SPSS, version 28.0; IBM SPSS Statistics for Windows, Armonk, NY).

Ethical considerations

This study was approved by the Ethics Research Committees of King Khalid Hospital (Reference: NAJ-IRB2024-076). The ethics committee granted a waiver of written consent due to high local illiteracy rates. Verbal informed consent was obtained from all participants using standardized scripts in Najrani Arabic. Data confidentiality was ensured through de-identification and secure separation of identifiers from study data.

## Results

Cohort characteristics

A total of 109 patients were included in the final analysis. Baseline sociodemographic and clinical characteristics are summarized in Table 1. The mean age of the cohort was 47.1 years (standard deviation ±17.2). The majority of participants were female (n=60, 55.0%), Saudi nationals (n=103, 94.5%), and married (n=89, 81.7%). Educational attainment was diverse, with the largest subgroup having completed secondary school (n=32, 29.4%). Most patients resided in urban areas (n=65, 59.6%) and were unemployed, retired, or homemakers (n=86, 78.9%). Clinically, 38 patients (34.9%) had three or more (≥3) comorbidities, and 46 patients (42.2%) were prescribed at least one cardiac-specific medication.

**Table 1:** Baseline sociodemographic and clinical characteristics of the study cohort (N=109). Data are presented as Mean ± Standard Deviation or n (%).

Characteristic	Category	n (%)
Age (years), Mean ± SD		47.1 ± 17.2
Gender	Male	49 (45.0)
	Female	60 (55.0)
Nationality	Saudi	103 (94.5)
	Non-Saudi	6 (5.5)
Marital Status	Married	89 (81.7)
	Single	16 (14.7)
	Widowed	4 (3.7)
Education Level	Cannot read/write	24 (22.0)
	Grade 1-9	28 (25.7)
	Secondary School	32 (29.4)
	University/College	31 (28.4)
Residence	Urban	65 (59.6)
	Rural	44 (40.4)
Employment Status	Employed	23 (21.1)
	Unemployed/Other	86 (78.9)
Number of Comorbidities	0	34 (31.2)
	1-2	37 (33.9)
	≥3	38 (34.9)
On Cardiac Medication	Yes	46 (42.2)
	No	63 (57.8)

Patient satisfaction across domains

Patient satisfaction was assessed across 12 domains. As indicated in Table 2, the overall satisfaction levels were high. The domains of "Confidentiality & Privacy" (n=85, 78.0%), "Ease of Use" (n=81, 74.3%), and "Overall Satisfaction" (n=80, 73.4%) received the highest proportions of "Excellent" ratings. In contrast, "Waiting Time" (n=52, 47.7%) and "Cost-Effectiveness" (n=57, 52.3%) had the lowest proportions of "Excellent" ratings. When "Excellent" and "Good" responses were combined, 90% or more of participants rated 10 of the 12 domains positively; the exceptions were Waiting Time (89.9%) and Cost-Effectiveness (88.1%).

**Table 2:** Patient satisfaction ratings across telemedicine domains (N=109). Data are presented as n (%).

Domain	Excellent	Good	Fair	Poor
Technical Quality (Audio/Video)	75 (68.8)	27 (24.8)	5 (4.6)	2 (1.8)
Ease of Use	81 (74.3)	23 (21.1)	4 (3.7)	1 (0.9)
Waiting Time	52 (47.7)	41 (37.6)	12 (11.0)	4 (3.7)
Duration of Consultation	68 (62.4)	33 (30.3)	6 (5.5)	2 (1.8)
Doctor's Explanation	77 (70.6)	26 (23.9)	4 (3.7)	2 (1.8)
Doctor's Attention	80 (73.4)	24 (22.0)	4 (3.7)	1 (0.9)
Treatment Plan Clarity	74 (67.9)	28 (25.7)	5 (4.6)	2 (1.8)
Confidentiality & Privacy	85 (78.0)	19 (17.4)	4 (3.7)	1 (0.9)
Convenience	79 (72.5)	25 (22.9)	4 (3.7)	1 (0.9)
Cost-Effectiveness	57 (52.3)	39 (35.8)	10 (9.2)	3 (2.8)
Willingness for Future Use	80 (73.4)	24 (22.0)	4 (3.7)	1 (0.9)
Overall Satisfaction	80 (73.4)	24 (22.0)	4 (3.7)	1 (0.9)

Factors associated with increased overall patient satisfaction

A multivariable logistic regression model was used to identify independent predictors of high overall satisfaction, defined as a composite score at or above the 75th percentile. Variables included in the initial model were age, gender, education (dichotomized as higher (secondary/university) vs. lower (none/primary)), residence, employment, number of comorbidities (dichotomized as <3 vs. ≥3), and use of cardiac medications. The final model was derived via backward elimination.

As shown in Table 3, two statistically significant independent predictors were identified. Higher educational attainment (secondary school or university level) was significantly associated with high satisfaction; individuals in this group had 3.1 times higher odds of reporting high satisfaction compared to those with lower or no formal education (adjusted odds ratio (aOR): 3.1, 95% CI: 1.2-8.0; p=0.019). Comorbidity burden was also a significant predictor; individuals having fewer than three comorbidities exhibited 2.8 times higher odds of high satisfaction compared to those with three or more comorbidities (aOR: 2.8, 95% CI: 1.1-7.3; p=0.034). It is important to note that the comorbidity variable represents a count of conditions rather than a weighted measure of clinical complexity.

**Table 3:** Results of multivariable logistic regression identifying predictors of high overall satisfaction. *Statistically significant (p < 0.05) Abbreviations: OR, Odds Ratio; CI, Confidence Interval; Sec, Secondary School; Uni, University/College

Predictor	Category	Unadjusted OR (95% CI)	p-value	Adjusted OR (95% CI)	p-value
Age	Per 1-year increase	1.00 (0.98-1.02)	0.899	1.00 (0.97-1.03)	0.891
Gender	Female vs. Male	1.3 (0.6-2.7)	0.501	1.4 (0.6-3.4)	0.442
Education	Higher (Sec/Uni) vs. Lower	2.6 (1.2-5.7)	0.017*	3.1 (1.2-8.0)	0.019*
Residence	Urban vs. Rural	0.8 (0.4-1.7)	0.601	0.8 (0.3-2.0)	0.596
Employment	Employed vs. Not	1.4 (0.6-3.4)	0.439	1.5 (0.5-4.3)	0.477
Comorbidities	<3 vs. ≥3	2.5 (1.2-5.4)	0.018*	2.8 (1.1-7.3)	0.034*
Cardiac Meds	Yes vs. No	0.9 (0.4-1.8)	0.705	0.9 (0.4-2.2)	0.874

Age, gender, residence, employment status, and use of cardiac medications were not significant predictors in the final model. Although trends suggested higher satisfaction among younger, female, and urban-dwelling patients, these were not statistically significant.

## Discussion

This study evaluated patient satisfaction with virtual cardiac consultations at King Khalid Hospital in Najran, Saudi Arabia, and examined demographic, clinical, and technical factors influencing satisfaction. The findings indicated a high level of satisfaction among patients regarding technical quality, provider communication, and appointment organization. Two principal outcomes emerged: overall patient satisfaction was exceptionally high, supporting the acceptability of this telemedicine model, and higher educational attainment and a lower comorbidity burden were identified as independent predictors of satisfaction. The absence of significant associations with age, gender, or rural versus urban residence suggests that telemedicine is broadly applicable across these demographic groups in this context.

The present findings, which demonstrate high patient satisfaction, align with and extend the growing body of evidence supporting the efficacy of telemedicine across diverse healthcare contexts. Internationally, studies conducted in Italy, the United Kingdom, and the United States have consistently identified user-friendly platforms and remote monitoring capabilities as fundamental drivers of positive patient experiences [17-19]. It is important to note, however, that reported satisfaction levels and their determinants show considerable heterogeneity across studies, influenced by differences in methodology, healthcare systems, and cultural contexts, which may limit direct generalizability. This international consensus is further supported by recent systematic evidence that highlights healthcare professionals' digital competence as crucial for successful telemedicine implementation, particularly their ability to establish reciprocal relationships and support patient self-management in digital environments [20]. In the Saudi context, these results corroborate earlier national findings, including Abdel Nasser et al.'s observation that 84.9% of patients found telemedicine facilitated healthcare access, with particular appreciation for the ease of registration and freedom of communication [4]. Similarly, Albagmi et al. documented generally high satisfaction levels among Saudi patients and practitioners, although they noted demographic influences that were not replicated in the more specialized cardiac cohort examined in the present study [2]. The convergence of these international and local findings underscores telemedicine's evolving role beyond crisis management toward sustainable healthcare delivery while highlighting the persistent importance of technological accessibility and professional digital competency across diverse clinical settings, including specialized cardiac care.

Multiple predictors of telemedicine satisfaction have been identified across various populations and healthcare settings, with significant variation influenced by cultural, technological, and clinical factors [21-23]. The present study adds to this body of evidence by identifying higher educational attainment and lower comorbidity burden as independent predictors within the Saudi cardiac telemedicine context. The strong association between education and satisfaction emphasizes digital literacy as a key social determinant of health, aligning with systematic reviews that cite education as a consistent barrier to telemedicine adoption across populations [11,15,20]. It is crucial to interpret this association carefully; higher educational attainment was strongly associated with higher satisfaction, likely serving as a proxy for greater digital literacy and comfort with technology, rather than being a direct causal determinant. Individuals with higher education are more likely to demonstrate confidence and technical proficiency in using digital platforms, which facilitates more effective healthcare interactions [24]. This result underscores the ongoing "digital divide," where technological progress may unintentionally widen health disparities in the absence of targeted support.

Furthermore, this finding must be interpreted within the robust framework of Social Determinants of Health (SDOH). Educational attainment is a cornerstone SDOH, intricately linked to health literacy and socioeconomic stability, which are fundamental drivers of cardiovascular risk and outcomes [4,5,7,21]. Therefore, patients with lower educational attainment may face a compounded burden: higher baseline cardiovascular risk due to socioeconomic factors, and potentially reduced ability to access or benefit from digital health innovations designed to manage that risk. Similarly, a higher comorbidity burden may reflect the cumulative impact of adverse SDOH [7,11]. Consequently, the association between lower education/comorbidity and reduced satisfaction underscores a critical risk: that telemedicine, without equity-focused strategies, could exacerbate existing health inequities.

The negative association between comorbidity burden and satisfaction highlights critical considerations regarding the clinical suitability of virtual care for complex patients. This association likely stems from patients with multiple conditions perceiving the virtual format as less adequate for comprehensive assessment, given its inherent lack of capacity for physical examination and nuanced bedside evaluation. This can be particularly salient for cardiac patients requiring close monitoring of heart failure signs, arrhythmia risk, or medication side effects, potentially leading to perceptions of less comprehensive care compared to in-person visits [20,25].

The analysis demonstrated that traditional demographic variables, such as age, gender, residence, employment status, and use of cardiac medications, did not significantly predict patient satisfaction within the telecardiology model. While trends indicated higher satisfaction among younger, female, and urban-dwelling patients, these associations were not statistically significant. The lack of a significant relationship with age or rural residence contrasts with previous telemedicine studies [26,27]. This discrepancy may be attributed to increased technology adoption across demographic groups and substantial national investments in rural broadband infrastructure, which underscore telemedicine's potential to reduce geographic healthcare disparities in well-resourced settings [28]. Enhanced familiarity and comfort with technology among diverse populations may have diminished the anticipated impact of demographic factors on telemedicine satisfaction [4]. However, these findings should be interpreted with caution, as the limited sample size may have reduced the statistical power to detect such associations. Future multicenter studies with larger cohorts are necessary to confirm these trends. Furthermore, the cohort's limited age range, with a predominance of middle-aged patients, may have contributed to the non-significant finding, as the influence of age on satisfaction has been more pronounced in studies with greater representation of older adults [26].

These findings are consistent with the digital-complexity framework for telemedicine implementation, which asserts that successful adoption is primarily determined by technological accessibility and clinical appropriateness rather than demographic characteristics. The framework highlights the importance of enhancing digital literacy and customizing care pathways for complex cases as key factors for success [29]. Regional studies support this perspective. For instance, research conducted in Jeddah found that older adults and first-time users were at greater risk of lower satisfaction, mainly due to a preference for in-person communication [26]. Furthermore, an evaluation of the Sehaty mobile health application in Saudi Arabia identified technical, usability, and functionality challenges as the principal barriers, whereas demographic factors did not significantly influence user experience [28]. Financial cost was not a significant barrier, likely due to government subsidies aligned with Vision 2030 objectives [30]. Nevertheless, time-related inefficiencies were reported, aligning with evidence that poorly designed applications can impede effective care management [3,31]. Overall, these findings indicate that addressing interconnected barriers requires a comprehensive strategy focused on technical reliability and user-centered design to optimize telemedicine adoption.

Despite our finding that rural residence was not a significant predictor of satisfaction in this cohort, it is widely recognized that patients in rural areas can face significant barriers to telecardiology engagement, primarily due to unreliable internet connectivity and limited technological resources [27,32]. While national infrastructure investments may mitigate these in some settings, such limitations can compromise audio-visual quality and negatively impact the experience where they persist. This digital divide can be compounded by lower health and digital literacy in rural populations, further challenging equitable access [4].

Operational and clinical challenges significantly influence patient satisfaction. Technical difficulties, missed appointments, and communication failures underscore the need for reliable telemedicine platforms and robust administrative support. Moreover, the limitations of virtual consultations, such as the inability to conduct physical examinations, may diminish clinical confidence and raise concerns regarding the comprehensiveness of care. These challenges support the implementation of hybrid care models that integrate telehealth with regular in-person assessments. The effectiveness of these models can be further enhanced by incorporating remote monitoring technologies, including wearable ECGs and blood pressure monitors, to support comprehensive clinical decision-making and patient management [5,27].

Study limitations

Several limitations should be considered. The cross-sectional, single-center design limits causal inference and generalizability. Potential selection bias, social desirability bias, and a ceiling effect due to high baseline satisfaction may have inflated ratings and limited variability. Methodologically, the use of an arbitrary percentile cutoff and a composite Likert-scale score represents a simplification. Furthermore, while education level was a significant predictor, it served as a proxy for digital literacy, which was not directly measured, limiting causal interpretation. Additionally, the comorbidity variable reflects a count rather than a weighted measure of complexity. It is possible that our study was underpowered to detect a smaller effect size for variables such as age and residence. Alternatively, it may genuinely indicate that in the specific context of Najran, these are not primary drivers of satisfaction, a finding that should be investigated in larger, multi-center studies across the Kingdom to confirm its generalizability. We invite collaborators from multiple centers to replicate this study to further validate our findings, particularly regarding the impact of rural residency on patient satisfaction.

## Conclusions

This study demonstrates that cardiac telemedicine services in Najran, Saudi Arabia, achieve high levels of patient satisfaction, supporting their acceptability and value as a sustainable element of contemporary healthcare delivery. It provides one of the first patient-centered evaluations of a dedicated cardiac telemedicine service in a non-urban Saudi region, focusing on predictors of satisfaction within a specialized clinical context - an underexplored area in the national literature. The widespread acceptance across age, gender, and residential location underscores this model's potential to expand access to specialized cardiac care throughout the region.

Nevertheless, the identification of patients with lower educational attainment and those with multiple comorbidities as significant predictors of reduced satisfaction exposes critical vulnerabilities. These results highlight a digital divide in which socioeconomic, educational, and clinical complexity factors influence the quality of the patient experience within digital health. To ensure that the benefits of telemedicine lead to equitable health outcomes, a proactive, targeted approach is required. Future strategies should extend beyond technological implementation to incorporate comprehensive support systems. This includes digital literacy initiatives and simplified user interfaces for patients with lower educational attainment, as well as tailored, integrated care pathways for those managing multiple chronic conditions. By deliberately designing telemedicine services that are both technologically advanced and broadly accessible, healthcare systems in Saudi Arabia and comparable settings can ensure these innovations reduce, rather than exacerbate, existing health disparities.
